# Supplementary material for: Splice-Junction-Based Mapping of Alternative Isoforms in the Human Proteome
Source: Cell Rep. Author manuscript; Available in PMC 2020 Jan 15. (PMC6961840; doi:10.1016/j.celrep.2019.11.026)
Supplement: 3 [file NIHMS1546469-supplement-3.zip › DF2/PXD0010154/AdrenalGland-187-O75390-TVVGQITVDMGIR.pdf]

A

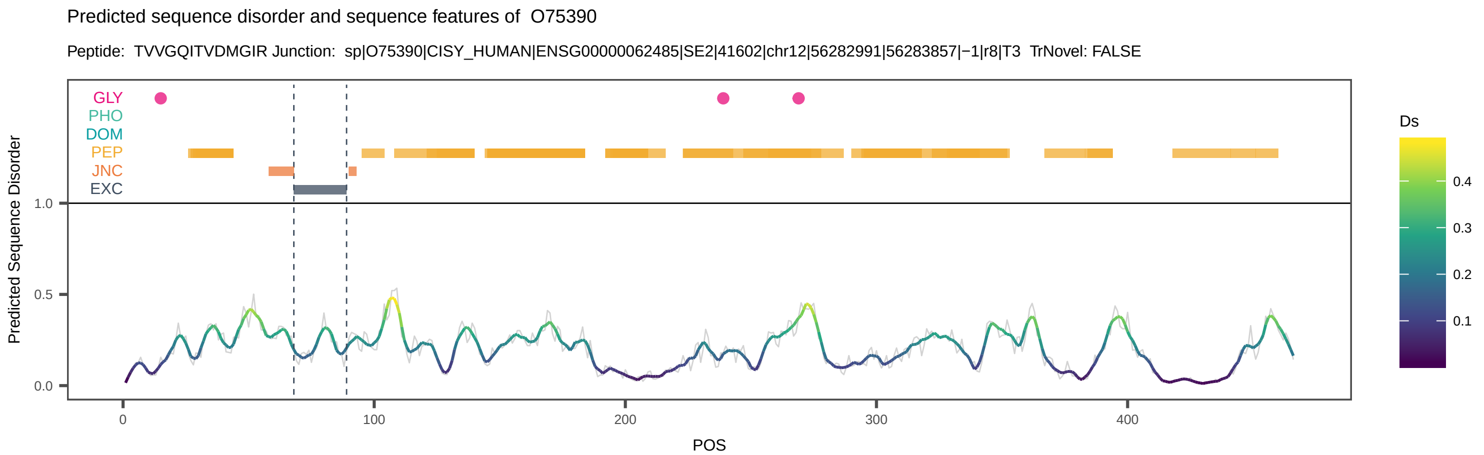
Predicted sequence disorder and sequence features of O75390
Peptide: TVVGQITVDMGIR Junction: sp|O75390|CISY_HUMAN|ENSG00000062485|SE2|41602|chr12|56282991|56283857|−1|r8|T3 TrNovel: FALSE
GLY
PHO
DOM
PEP
JNC
EXC
Predicted Sequence Disorder
1.0
0.5
0.0
0
100
200
300
400
POS
Ds
0.4
0.3
0.2
0.1

B

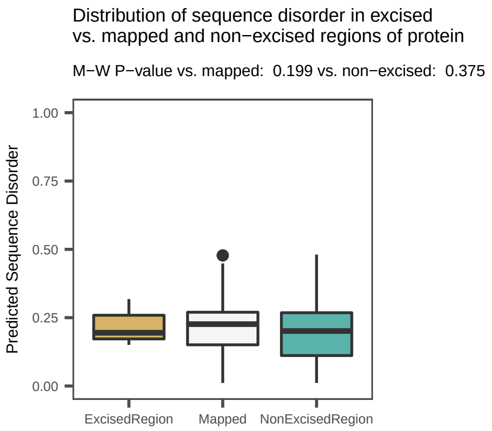
Distribution of sequence disorder in excised
vs. mapped and non−excised regions of protein
M−W P−value vs. mapped: 0.199 vs. non−excised: 0.375
Predicted Sequence Disorder
1.00
0.75
0.50
0.25
0.00
ExcisedRegion
Mapped
NonExcisedRegion

C
